# Immunomodulatory Effects of Diterpenes and Their Derivatives Through NLRP3 Inflammasome Pathway: A Review

**DOI:** 10.3389/fimmu.2020.572136

**Published:** 2020-09-25

**Authors:** Muhammad Torequl Islam, Sanaa K. Bardaweel, Mohammad S. Mubarak, Wojciech Koch, Katarzyna Gaweł-Beben, Beata Antosiewicz, Javad Sharifi-Rad

**Affiliations:** ^1^Laboratory of Theoretical and Computational Biophysics, Ton Duc Thang University, Ho Chi Minh City, Vietnam; ^2^Faculty of Pharmacy, Ton Duc Thang University, Ho Chi Minh City, Vietnam; ^3^Department of Pharmaceutical Sciences, School of Pharmacy, The University of Jordan, Amman, Jordan; ^4^Department of Chemistry, The University of Jordan, Amman, Jordan; ^5^Chair and Department of Food and Nutrition, Medical University of Lublin, Lublin, Poland; ^6^Department of Cosmetology, University of Information Technology and Management in Rzeszów, Rzeszów, Poland; ^7^Zabol Medicinal Plants Research Center, Zabol University of Medical Sciences, Zabol, Iran

**Keywords:** diterpenes, inflammation, mitochondrial dysfunction, NLRP3, mechanism of action

## Abstract

Nucleotide-binding oligomerization domain-like receptor family pyrin domain-containing protein (NLRP) inflammasomes are involved in the molecular pathogenesis of many diseases and disorders. Among NLRPs, the NLRP3 (in humans encoded by the *NLRP3* gene) is expressed predominantly in macrophages as a component of the inflammasome and is associated with many diseases, including gout, type 2 diabetes, multiple sclerosis, atherosclerosis, and neurological diseases and disorders. Diterpenes containing repeated isoprenoid units in their structure are a member of some essential oils that possess diverse biological activities and are becoming a landmark in the field of drug discovery and development. This review sketches a current scenario of diterpenes or their derivatives acting through NLRPs, especially NLRP3-associated pathways with anti-inflammatory effects. For this, a literature survey on the subject has been undertaken using a number of known databases with specific keywords. Findings from the aforementioned databases suggest that diterpenes and their derivatives can exert anti-inflammatory effects *via* NLRPs-related pathways. Andrographolide, triptolide, kaurenoic acid, carnosic acid, oridonin, teuvincenone F, and some derivatives of tanshinone IIA and phorbol have been found to act through NLRP3 inflammasome pathways. In conclusion, diterpenes and their derivatives could be one of the promising compounds for the treatment of NLRP3-mediated inflammatory diseases and disorders.

## Introduction

Inflammation, the body's natural response to harmful stimuli, arises in tissues due to traumatic, infectious, post-ischemic, toxic, or autoimmune injuries. To restore the normal tissue functions during the harmful inflammatory conditions, clearance of inflammatory cells, along with pro-inflammatory signaling pathways, are required (1). The nucleotide-binding oligomerization domain-like receptor family pyrin domain-containing proteins (NLRPs), specifically NLRP3, a cytosolic innate immune signaling receptor, have been detected at elevated levels in several inflammatory diseases, such as chronic infantile neurological cutaneous and articular (CINCA) syndrome, familial cold auto-inflammatory syndrome (FCAS), keratoendotheliitis fugax hereditaria, Muckle–Wells syndrome (MWS), neonatal onset multisystem inflammatory disease (NOMID) (2, 3), and familial Mediterranean fever (4). In addition, NLRP3 has been associated with gout (5), type 2 diabetes, multiple sclerosis, atherosclerosis (6), and Alzheimer's, Parkinson's, and prion diseases (7–9). Furthermore, the deregulation of NLRP3 is associated with carcinogenesis, such as hepatocellular carcinoma (10, 11). Thus, the pharmacological inhibition of NLRP3 activity may possibly present a promising approach for the management and cure of inflammatory and other related diseases in both humans and animals (12).

Non-steroidal anti-inflammatory drugs (NSAIDs) are the most used medications for the treatment of inflammation and related diseases. However, NSAIDs cause many adverse effects, including gastrointestinal complications, immunodeficiency, and humoral disturbances, which may limit their use in certain patients (1). The natural products or their derivatives are comparatively safe; therefore, these can be considered as potential platforms for the discovery with anti-inflammatory properties (13). Among plant metabolites, essential oils (EOs) have gained much attention due to their possible biomedical applications. Diterpenes, a category of components present in some EOs, which contain repeated isoprenoid units in their structure and modulate diverse biological activities, are becoming a landmark in the field of EOs research (14–16).

Recently, Tricarico and coworkers suggested that isoprenoids may exert their effect by modulating different pathways in the biological systems primarily by increasing the expression of various markers for apoptosis, cytokines, mitochondrial dysfunction, NLRP3, as well as nitric oxide (NO) (17). Based on the preceding discussion and the importance of the NLRP3 inflammasome in the pathogenesis of gout and neuroinflammation occurring in protein misfolding diseases, such as Alzheimer's and Parkinson's, among others, in this review, we have sketched a current scenario of diterpenes or their derivatives acting through NLRPs, especially NLRP3-associated pathways with anti-inflammatory effects.

## Search Strategy

A literature search was conducted through June 2020 by means of a number of well-known databases, such as Google Scholar, PubMed, Scopus, ScienceDirect, Web of Science, the American Chemical Society, and ClinicalTrials.gov, using the keywords “diterpenes or diterpenoids” and “diterpene derivatives,” combined with “NLRP3 or NLRP3 inflammasome.” No language restrictions were imposed. The following are the inclusion and exclusion criteria of this study.

### Inclusion Criteria

- Studies with diterpenes and their derivatives or preparations acting against NLRP3;
- Studies (*in vitro, ex vivo*, or *in vivo*) with or without using experimental animals, including humans and their derived tissue and cells;
- Studies that utilized single and/or multiple cell lines or animals;
- Studies with diterpenes isolated from plants/other natural sources;
- Diterpenes or their derivatives joint effects with other substances (including diterpenes, drugs, or chemicals/biochemicals);
- Studies with or without proposing activity mechanisms.

### Exclusion Criteria

- Studies with extracts without mentioning diterpene contents;
- Studies with essential oils other than diterpenes;
- Duplication of data and titles and/or abstracts not meeting the inclusion criteria;
- Diterpenes acting against NLRPs other than NLRP3;
- Diterpenes with other studies uncovering the current topic.

## NLRP3-Mediated Inflammatory Pathways

Macrophages stimulated by priming stimuli, such as ligands for toll-like receptors (TLRs), NOD-like receptors (NLRs) (e.g., NOD-1 and−2), or cytokine receptors, can activate nuclear factor kappa B (NF-κB). The signaling molecules MyD88 and TRIF of the NF-κB signaling pathway regulate the induction of NLRP3 and pro-interleukin 1 beta (IL-1β) in response to TLR ligands (18). However, the apoptotic signaling molecules caspase-8 and fas-associated protein with death domain (FADD) are also required for the induction of the NLRP3 during the priming process (19). Priming signals triggering c-Jun N-terminal kinase 1 (JNK1)-mediated NLRP3 phosphorylation are a critical event for NLRP3 self-association and inflammasome activation (20).

**Figure 1:**
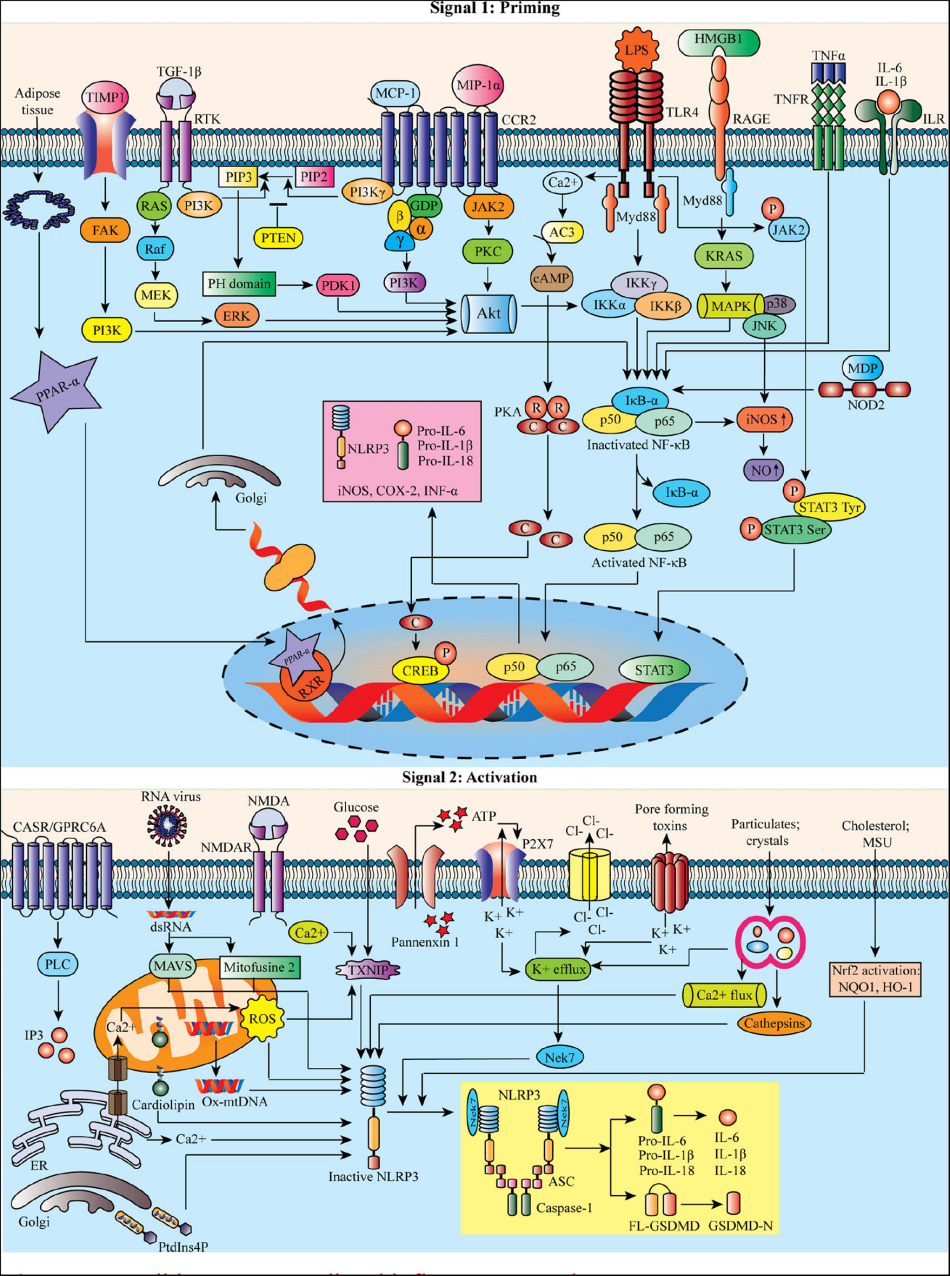
Possible NLRP3-mediated inflammatory pathways.

Reactive oxygen species (ROS) are proposed as a common signal for NLRP3 inflammasome (21). Mitochondrial dysfunction and mitochondrial ROS (mtROS) production are dispensable in NLRP3 inflammasome activation (22). Besides this, the mitochondria can co-localize with the NLRP3 inflammasome. Still, the mechanism associated with lysosomal disruption to NLRP3 inflammasome activation remains unclear. It has been proposed that the active lysosomal enzymes released into the cytosol may trigger NLRP3 inflammasome activation after the phagocytosis of a particulate matter. Lipopolysaccharide (LPS) can result in NLRP3-mediated IL-1β processing and secretion in a P2X purinoceptor 7 (P2X7) independent manner (23). Figure 1 represents the possible NLRP3-mediated inflammatory pathways.

## Diterpenes Modulate NLRP3 Inflammasome Pathways: Literature Findings

**Figure 2:**
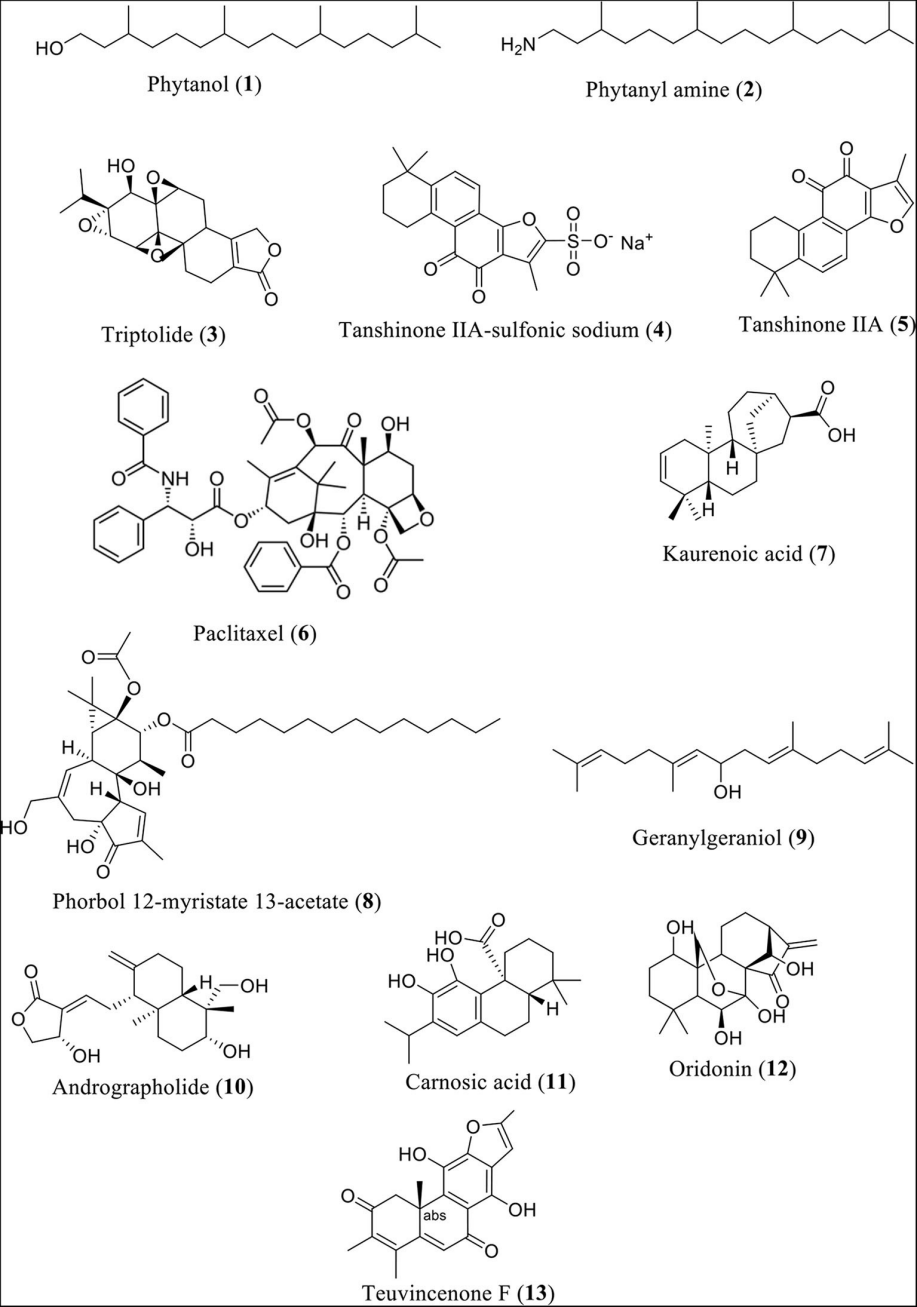
Structures of some diterpenes or diterpenoids and their derivatives.

Research findings revealed that phytanol (Compound 1) and phytanyl amine (Compound 2) reduce the expression of NLRP3, along with interleukin (IL)-6, monocyte chemoattractant protein-1 (MCP-1), keratinocyte chemoattractant (KC), macrophage inflammatory protein 1 (MIP-1), and lipopolysaccharide-induced CXC chemokine (LIX, also GARG-8) (24). Additionally, reduction of the release of B-lymphocyte chemoattractant (BLC), T-cell activation-3 chemokines tricarboxylic acid (TCA), IL-4, IL-12, and tissue inhibitor of metalloproteinase-1 (TIMP-1) in BALB/c mice was observed. In a similar fashion, the heartwood of Taiwan fir (diterpene content 0.9%) was found to lower NLRP3 inflammasome-derived IL-lβ secretion induced by LPS and adenosine triphosphate in mouse macrophages (25). It also lowered the levels of IL-lβ precursor and reduced the secretion of NLRP3 inflammasome-derived IL-lβ and adenosine triphosphate (26). Figure 2 shows the structures of diterpenes or diterpenoids and their derivatives involved in this investigation.

Triptolide (Compound 3) significantly reduced the serum levels of IL-1β and IL-18, along with NLRP3 and toll-like receptor 4 (TLR4), expressions in female Sprague–Dawley rats (27). It may prevent the progression of immunoglobulin A (IgA) nephropathy by ameliorating the inflammasome-mediated pro-inflammatory cytokine production, thus providing new insight into the treatment of this disease (28). Moreover, it exerted a cardio-protective effect in C57/BL6 mice *via* the attenuation of transverse aortic constriction-induced myocardial remodeling. This effect (a) enhanced cardiac diastolic and systolic functions, (b) activated the pro-fibrotic tumor growth factor 1 beta (TGF-1β) pathway, (c) suppressed the NLRP3 inflammasome and inflammatory mediators of IL-1β, IL-18, MCP-1, and vascular cell adhesion molecule-1 (VCAM-1), and (d) dose-dependently inhibited macrophage infiltration (29). In a recent study, it has been found to inhibit the NLRP3–TGF1β-Smad pathway, suggesting that this compound might be an alternative option for cardiac fibrosis *via* targeting the NLRP3 inflammasome (30). Furthermore, it down-regulated NLRP3 by targeting hsa-miR-20b in male C57BL/6 mice and in THP-1 cells (31).

Tanshinone IIA (Compound 5) at 0–2.5 μg/ml exerted a protective effect by down-regulating NLRP3, caspase-1 (CASP1), IL-1β, and IL-18 in BV-2 cells (32). The male Sprague–Dawley rats (*n* = 15) treated with this compound at 10 mg/kg/day (i.p.) for 1 week down-regulated tumor necrosis factor alpha (TNF-α), IL-4, TLR4, MyD88, NLRP3, and NF-κB p65 expression levels and up-regulated IL-10, tumor growth factor-beta (TGF-β), phosphatase and tensin homolog (PTEN), phosphoinositide 3-kinase (PI3K), and AKT (also called protein kinase B) phosphorylation levels (33). Liu and coworkers have recently shown that in A549 cells, paclitaxel (Compound 6) can cause apoptotic cell death by ROS-induced caspase-3 activation *via* the activated apoptosis signal-regulating kinase 1 (ASK1)/p38 mitogen-activated protein kinase (MAPK) signaling pathway (25). On the other hand, sodium tanshinone IIA sulfonate (Compound 4) reduced the overproduction and overexpression of cardiac ROS and thioredoxin-interacting protein (TXNIP), respectively, through diminishing the Janus kinase 2 (JAK2)–signal transducer and activator of transcription 3 (STAT3)/insulin signaling/peroxisome proliferator-activated receptor-α (PPAR-α) pathway in Beagle dogs (34). In another study, kaurenoic acid (Compound 7) restored the production of nitric oxide (NO) in a constitutive NO synthase- (cNOS-) dependent fashion, increased the formation of IL-1β, and elevated the expression of NLRP12 in *Leishmania amazonensis* infected BALB/c mice (35). Phorbol myristate acetate (Compound 8) at 100 nM down-regulated the mRNA expression of CASP1, IL-1β, IL-18, myeloid leukemia cell differentiation protein 1 (MCL1), NLRP3, as well as PYCARD expression in BALB/cMlac mice neutrophil cells (36). In contrast, geranylgeraniol (Compound 9), diterpenoid alcohol, at 50 μM was found to reduce the expression of *NLRP3* gene and mitochondrial dysfunction-linked programmed cell death in a neuronal cell line (Daoy) (37).

Andrographolide (Compound 10), the bitter diterpene lactone, at 7.5 and 15 mg/kg exerted an anti-colitis and anti-tumor effect by lowering the expression of cleaved CASP1, IL-1β, and mitochondrial membrane potential collapse *via* the PIK3CA–AKT1–MTOR–RPS6KB1 pathway. It additionally increased the disruption of NLRP3–PYCARD–CASP1 complex formation and mitophagy in macrophages, inactivated the NLRP3 inflammasome, and induced autophagy in mice (38). In another study, andrographolide was also seen to down-regulate the expression of TNF-α, IL-1β, and NLRP3 and suppressed ROS-mediated NF-κB expression in ovalbumin (OVA)-induced female C57/BL6 mice and BMDM cells (39). Furthermore, a recent study in mice HepG2 cells demonstrated that this diterpene lactone exhibits a hepatoprotective effect in choline-deficient, l-amino acid defined (CDAA)-fed mice; this was accomplished by reducing the hepatic inflammation and fibrosis. It also lowered the hepatic mRNA levels of both pro-inflammatory and pro-fibrotic genes, as well as hepatic macrophage infiltration, and reduced the expression of inflammasome genes, IL-1β expression through NF-κB inhibitory pathway, and inflammasome disassembly (40). In addition, research findings revealed that Compound 10 (a) lowers the overexpression of LPS-induced high-mobility group protein 1 (HMGB1), TLR4, NF-κB, cyclooxygenase-2 (COX-2), inducible nitric oxide synthase (iNOS), and NLRP3, (b) suppresses the overexpression of microglial macrophage inflammatory protein 1-alpha (MIP-1α) and P2X7 receptor, along with its downstream signaling mediators, including CASP1 and mature IL-1β, and (c) modulates the expression of protein markers, such as protein kinase C (PKC), phosphorylated cAMP response elements (p-CREB), amyloid-beta (Aβ), amyloid precursor protein (APP), p-tau, synapsin, and PSD-95 in mouse glial cells (41).

Carnosic acid (Compound 11) lowered the expression of NLRP3 and CASP1, along with myeloperoxidase (MPO) levels, in male BALB/c mice. Additionally, it increased the level of nuclear factor erythroid 2 (Nrf2) and stopped the degradation of Nrf2 through a mechanism that involves impeding the interaction between Cullin3 and Keap1. It also increased the reduced glutathione (GSH) and superoxide dismutase (SOD) levels and caused a decrease in malondialdehyde (MDA) and iNOS levels in experimental animals (42). In wild-type C57BL/6 and myristoylated alanine-rich C-kinase substrate (MARCKS) deficient mice, this diterpene has been found to suppress the PI3K/AKT, NLRP3/NF-κB, and sterol regulatory element-binding protein 1 (SREBP-1c) signaling pathways (43). On the other hand, oridonin (Compound 12) exhibited an anti-inflammatory effect in mice and HEK-293T cells, through the interaction with the cysteine 279 of NLRP3 in NACHT domain, thereby blocking the interaction between NLRP3 and NEK7 and inhibiting NLRP3 inflammasome formation and activation. Oridonin also reduced peritonitis, gouty arthritis, and type 2 diabetes in experimental animals by inhibiting NLRP3 activation (27). Similarly, teuvincenone F (Compound 13) attenuated the K63-linked ubiquitination of NF-κB-essential modulator inhibitor of nuclear factor kappa-B kinase gamma (IKKγ) and inhibited the mRNA expression of IL-1β, IL-6, IL-1β/IL-18, and TNF-α maturation in wild-type C57BL/6 mouse macrophages (44). However, a study by Huang et al. (26) suggested that phorbol 12-myristate 13-acetate induced human monocytic THP-1 cells and increased NLRP3 inflammasome expression by up-regulating the TLR4/MyD88/NF-κB signaling pathway. In a similar fashion, paclitaxel caused mitochondrial damage and overproduction of ROS, along with activation of NLRP3 and neuropathic pain in male adult Sprague–Dawley rats (45). Listed in Table 1 are diterpenes and their derivatives' biological effects through NLRP3 inflammasome.

**Table 1:** Summary of bioactivities of diterpenes and/or their derivatives: test dose/conc., test system(s), and possible NLRP3 inflammasome and its associated mechanisms.

**Diterpenes and/or their derivatives**	**Conc./dose (route of administration)/test system**	**NLRP3 and NLRP3-associated action mechanism**	**References**
Phytanol, phytanyl amine	Phytanol: 40 mg (i.p.) and phytanyl amine: 5 mg (i.p.) in immunoadjuvant effects in BALB/c mice	Immunostimulatory effect (decreases the expression of IL-6, MCP-1, KC, MIP-1, LIX, BLC, T-cell activation-3 chemokines TCA, IL-4, IL-12, and TIMP-1)	(16)
Triptolide	200 μg/kg/day (i.g.) in female Sprague–Dawley rats from 12 to 28 weeks (*n* = 15)	Anti-inflammatory effect (decreases the expression of IL-1β, IL-18, NLRP3, and TLR4)	(45)
	20 or 100 μg/kg/day (i.p.) in C57/BL6 mice (7–10)	Cardio-protective effect (attenuates transverse aortic constriction-induced myocardial remodeling and increases cardiac diastolic and systolic functions, TGF-β1 pathway, and pro-fibrotic genes. In a dose-dependent manner, it additionally lowers NLRP3 inflammasome and downstream of IL-1β, IL-18, MCP-1, VCAM-1, and macrophage infiltration	(46)
	*In vivo*: 0.0035 mg/ml (i.p.) in male C57BL/6 mice (*n* = 5) *In vitro*: 20 ng/ml in THP-1 cells	Prevention of osteoarthritis (decreases CASP1 and targeting hsa-miR-20b)	(27)
	10 μg/ml in mouse cardiac fibroblast cells	Reduced IL-1β maturation, MyD88-related phosphorylation of JNK, ERK1/2, and TGF-β1/Smad signaling, thereby decreasing collagen production, and inhibited NLRP3 expression and apoptosis-associated speck-like proteins containing a caspase recruitment domain (ASC)	(30)
Tanshinone IIA	10 mg/kg/day (i.p.) for 1 week in male Sprague–Dawley rats (n = 15)	Hepatoprotective effect (decreases the expression of TNF-α, IL-4, TLR-4, MyD88, and p-NF-κB p65 and increases the phosphorylation of IL-10, TGF-β, PTEN, PI3K, and AKT)	(34)
	0–2.5 μg/ml in BV-2 cells	Protective effect (decreases NLRP3, CASP1, IL-1β, and IL-18)	(31)
Sodium tanshinone IIA sulfonate	1.3, 2.6, and 5.2 mg/kg (i.v.) in Beagle dogs (*n* = 6)	Inhibition of myocardial inflammation and lipid accumulation (decreases ROS and TXNIP up-expression through an impairment of the JAK2–STAT3/insulin signaling/PPAR-α pathway)	(33)
Paclitaxel	0.5, 1, 5, 10, 50, and 100 μg/ml in A549 cells	Apoptotic cell death (decreases ROS-induced caspase-3 activation and increases the ASK1/p38 MAPK signal pathway)	(32)
	2 mg/kg (i.p.) for 7 days in male adult Sprague–Dawley rats	Neuropathic pain (increases mitochondrial damage and ROS production and activates NLRP3 inflammasome)	(44)
Kaurenoic acid [*ent*-kaur-16-en-19-oic acid]	50, 70, and 90 μM in *Leishmania amazonensis* infected BALB/c mice	Leishmanicidal activity (reestablishes the production of NO in a constitutive NO synthase- (cNOS-) dependent fashion and increases the expression of IL-1β and NLRP12)	(35)
Phorbol myristate acetate	100 nM in mouse [BALB/cMlac mice (*n* = 24)] neutrophil cells	Neuroprotective effect (decreases the mRNA expression of CASP1, IL-1β, IL-18, MCL1, and PYCARD)	(25)
Phorbol 12-myristate 13-acetate	Human monocytic THP-1 cells	Inflammatory effect (increases the expression of the NLRP3 inflammasome by up-regulation of the TLR4/MyD88/NF-κB signaling pathway)	(43)
Andrographolide	1 mg/kg (p.o.), 3-times/week) in mice (n = 7–10) and 20 and 50 M in HepG2 cells	Hepatoprotective effect (decreases inflammation, fibrosis, hepatic macrophage infiltration, hepatic mRNA levels of both pro-inflammatory and pro-fibrotic genes, expression of inflammasome genes, IL-1β expression through the NF-κB inhibitory pathway, and inflammasome disassembly)	(38)
	*In vivo*: 5 and 10 mg/kg (i.p. for 10–14 days) in OVA-induced female C57/BL6 mice (*n* = 8) *In vitro*: 30 μM in OVA-induced BMDM cells	Inhibition of lung injury (decreases the expression of TNF-α, IL-1β, and ROS-mediated NF-κB)	(37)
	1 μg/ml in mouse glial cells	Neuropharmacological effect (decreases HMGB1, TLR4, NF-κB, COX-2, iNOS, MIP-1α, P2X7, CASP1, and mature IL-1β and modulates the expression of protein markers, such as PKC, p-CREB, amyloid-beta, APP, p-tau, synapsin, and PSD-95)	(39)
	7.5 and 15 mg/kg (i.g.) mice (*n* = 6)	Anti-colitis and anti-tumor effect (decrease the expression of cleaved CASP1, IL-lβ, mitochondrial membrane potential collapse, and PI3KCA–AKT1–mTOR–RPS6KB1 pathway; increases the disruption of NLRP3–PYCARD–CASP1 complex assembly and mitophagy in macrophages; inactivates the NLRP3 inflammasome; and induces autophagy)	(36)
Carnosic acid	50 and 100 mg/kg (p.o.) for 3 days in male BALB/c mice (n = 8)	Inhibition of acute colitis (decreases CASP1 expression and MPO, MDA, and iNOS levels; increases Nrf2 expression; prevents the degradation of Nrf2 *via* ubiquitination by blocking the interaction between Cullin3 and Keap1; and increases GSH and SOD levels)	(40)
	30 mg/kg (i.g.) in male wild-type C57BL/6 and MARCKS deficient mice (n = 15)	Non-alcoholic fatty liver disease (decreases PI3K/AKT, NLRP3/NF-κB, and SREBP-1c signaling pathways)	(41)
Oridonin	3 or 20 mg/kg (i.p.) once a day for 6 weeks in C57BL/6J and WT or Nlrp3^−/−^ mice (*n* = 6); 0.1–2 μM in HEK-293T cells	Anti-inflammatory effect (increases the interaction with cysteine 279 of NLRP3 in NACHT domain; blocks the interaction between NLRP3 and NEK7; inhibits NLRP3 inflammasome assembly and activation; reduces peritonitis, gouty arthritis, and type 2 diabetes; and inhibits NLRP3 activation)	(24)
Teuvincenone F	6.25, 12.25, and 25 μM in wild-type C57BL/6 mouse macrophages	Anti-inflammatory effect (decreases the K63-linked ubiquitination of NF-κB-essential modulator IKKγ and mRNA expression of IL-1β, IL-6, TNF-α, and IL-1β/IL-18 maturation)	(42)
Heartwood of Taiwan fir (diterpene content 0.9%)	LPS-activated macrophages	Anti-inflammatory effect (decreases NLRP3 inflammasome-derived IL-lβ secretion induced by LPS and ATP)	(47)
Geranylgeraniol	50 μM in a neuronal cell line (Daoy)	Neurological impairment (decreases *NLRP3* gene expression and mitochondrial dysfunction-linked programmed cell death)	(48)

## Discussion

The NLRP3 is activated by many and diverse stimuli, making it the most versatile and important clinically implicated inflammasome. The intracellular NLRP3 receptor might be able to detect its levels through direct interactions, although it is reported that NLRP3 responds to certain generic cellular stress signals induced by the multitude of pathogen-associated molecular patterns (PAMPs) and damage-associated molecular patterns (DAMPs) that trigger its activation (49). The high-mobility group box 1 (HMGB1) protein is also known for its activity, such as PAMP and DAMP components, which exaggerates immune stimulations at the time of tissue injury (50). Therefore, HMGB1 may be a potential therapeutic target in severe pulmonary inflammation, including coronavirus disease 2019 (Covid-19) caused by severe acute respiratory distress syndrome coronavirus 2 (SARS-CoV-2) (51). EOs are natural products with a complex composition. Terpenes are the most common class of chemical compounds present in EOs with diverse biological activities (52) and can be used as chemopreventive and therapeutic agents for treating various inflammatory diseases (53). Among terpenes, diterpenes and triterpenes are vastly studied due to their promising therapeutic benefits. Glycyrrhizin (also called glycyrrhizic acid), a triterpenoid saponin isolated, is used in Chinese Pharmacopeia (54). Recently, it has been demonstrated that this triterpene may be a hope in the Covid-19 pandemic (55). Diterpenes are often with the molecular formula C_20_H_32_, which is composed of repeated isoprene subunits. Diterpenes and their derivatives are known for their antimicrobial and anti-inflammatory activities (14). In this study, it has been observed that diterpenes and their derivatives have interacted with NLRP3 in a variety of ways. For an example, andrographolide has been seen to reduce neuroinflammation in mouse glial cells through the HMGB1 pathway (39).

For the activation of pro-CASP1, research findings showed that caspase activation and recruitment domains (CARDs) act as a platform termed the ASC speck (56). Along this line, active CASP1 initiates the activation and release of IL-1 proteins (57), whereas the activation of NLRP3 causes the release of some pro-inflammatory mediators, including IL-1β, IL-18, and high-mobility group protein B1 (HMGB1) (58–60). Triptolide (30, 45, 46), tanshinone IIA (31), phorbol myristate acetate (25), and teuvincenone F (42) have been seen to reduce both IL-1β and IL-18 expressions, whereas kaurenoic acid (35) and andrographolide (36–39) reduced the IL-1β expression in different test systems. Tanshinone IIA also attenuated ischemia/reperfusion injury (IRI) induced liver injury through down-regulating the HMGB1-TLR-4/NF-κB pathway in Kupffer cells (KCs) and activated the PTEN/PI3K/AKT pathway (29).

NACHT, LRR, and PYD domains-containing protein 12 (NLRP12), a member of the NLRs, which is known to interact with the apoptosis-linked speck-like protein containing a CARD (ASC), cause the activation of CASP1, resulting in IL-1β release (61). Recently, it has been reported that the NLRP12, through its binding to the hematopoiesis cell kinase, may impart an effect on the pathogenesis of acute myeloid leukemia (62). Kaurenoic acid was seen to exert leishmanicidal activity through triggering a NLRP12/IL-1β/cNOS/NO pathway (35).

On the other hand, IKKγ activates NF-κB. Furthermore, during the activation of NLRP3, transcription of the *NLRP3* gene occurs, which activates NF-κB, including TLRs and TNF receptors (18, 63). In a recent study, triptolide was found to down-regulate hsa-miR-20b by targeting the *NLRP3* gene in mice (33). Tanshinone IIA (34) and teuvincenone F (42) reduced TNF-α expression, whereas andrographolide was evident to down-regulate both NF-κB and TNF-α expressions (37–39, 41). Additionally, teuvincenone F modulated the IKKγ expression in mouse macrophages (42).

MyD88 was suggested to be responsible for NF-κB-dependent transcriptional priming (64). Triptolide (30), tanshinone IIA (34), and phorbol 12-myristate 13-acetate (43) were seen to act through this pathway. TLR4 stimulates NLRP3 inflammasome specific monocytes, which is alone sufficient to cause IL-1β and pro-IL-18 release (65). Triptolide (45), tanshinone IIA (34), phorbol 12-myristate 13-acetate (43), and andrographolide (39) were seen to act *via* this pathway. Moreover, p-CREB directly inhibits NF-κB activation by a mechanism that involves hindering the binding of CREB-binding protein to the NF-κB complex, thereby limiting pro-inflammatory responses and suggesting the induction of anti-apoptotic effect, along with proliferation and cell survival (66). Andrographolide was found to modulate the p-CREB expression in mouse glial cells (39). Therefore, the anti-inflammatory effects of the herbal contents and diterpenes (26, 28, 44, 67) may be related to their interaction potentials with NLRP3 by various pathways.

In the meantime, sterol regulatory element-binding proteins (SREBPs) are known to regulate lipid homeostasis and activate the transcription of genes encoding enzymes involved in the biosynthesis of compounds including cholesterol, triglycerides, phospholipids, and fatty acids. In this regard, SREBP-1c, one of the three SREBP isoforms, activates genes that are involved in fatty acid synthesis (68); this isoform can be activated by an AKT-dependent pathway (69). Similarly, research findings indicated that NLRP3 inflammasome complex/CASP1 triggers SREBP and promotes membrane biogenesis, resulting in host cell survival in response to some toxins (70). In addition, the hepatoprotective effect of tanshinone IIA (29) may be related to the inhibitory capacity of the NLRP3 inflammasome-induced inflammation cascade pathway, whereas for carnosic acid (43), it might be due to the inhibition capacity of SREBP-1c signaling pathways.

The opening of the P2X7 channel may lead to the accumulation of high levels of extracellular ATP (71) when there are dying cells in the vicinity of inflammasome-containing cells. Furthermore, aggregation of islet amyloid polypeptide (IAPP), a hormone secreted by β cells and Aβ plaques, may cause interaction with phosphorylated tau (p-tau) and damage to the neuronal structure and function, particularly synapses; this can lead to a cognitive decline in Alzheimer's patients (72–74). Andrographolide reduced P2X7, whereas it modulated p-tau expression in mouse glial cells (39). Moreover, mitochondrial dysfunction and production of mtROS are considered vital for the activation of NLRP3 inflammasome (75), whereas subsequent activation of STAT3 limits NLRP3 inflammasome priming (76). Interleukins from IL-12 family, such as IL-27, may enhance the LPS response in monocytes, in a STAT3-NF-κB-dependent manner through the up-regulation of TLR4 expression (77). Sodium tanshinone IIA sulfonate decreased ROS and TXNIP overexpression through an impairment of the JAK2–STAT3/insulin signaling/PPAR-α pathway (33).

The nuclear factor E2-related factor 2 (Nrf2) plays a regulatory role in the NLRP3 inflammasome. Nrf2 constantly degrades upon ubiquitination under normal conditions. These inflammasomes represent stress-induced protein complexes that are involved in acute and chronic inflammation by means of CASP1-mediated activation of pro-inflammatory cytokines (78). Carnosic acid increased Nrf2 expression and prevents the degradation of Nrf2 *via* ubiquitination by blocking the interaction between Cullin3 and Keap1 (40). In addition, intragraft KC expression, associated with NLRP3 inflammasomes, can be considered as a new therapeutic strategy in the treatment of liver graft injury (34). Moreover, PPARs, especially the PPARα, are needed to prevent excessive inflammatory responses, as it controls the inflammasome complex NLRP3 activation (79). TXNIP translocates to the mitochondria, along with NLRP3, and causes activation of the inflammasome, which is also responsible for endoplasmic reticulum (ER) stress in animals (80). Sodium tanshinone IIA sulfonate inhibited myocardial inflammation and lipid accumulation *via* decreasing TXNIP up-expression through an impairment of the JAK2–STAT3/insulin signaling/PPAR-α pathway (33). In this regard, the PI3K–AKT pathway is responsible for the ER-mediated Ca^2+^ release (81). The voltage-dependent anion channel is involved in the transport of Ca^2+^ to the mitochondria, which accelerates ROS production and inflammasome activation (82). Tanshinone IIA (34), andrographolide (36), and carnosic acid (41) were seen to act through the PI3K–AKT-dependent pathway. Recent research findings indicated that inflammasome-independent NLRP3 enhances TGF-β1 signaling in certain tissues, including kidney epithelium and cardiac fibroblasts (83, 84). Tanshinone IIA increased TGF-β1 expression (34), whereas triptolide reduced TGF-β1/Smad signaling (30) in experimental animals.

In primary human macrophages, p38δ MAPK has been recognized as a regulator of NLRP3 inflammasome activation, which might be one of the potential targets for the treatment of atherosclerotic inflammation (85). On the other hand, TIMP-1 is a glycoprotein expressed in several tissues in our body. TIMP-1 plays a role in wound healing (86) and pregnancy (87). The dysregulated activity of TIMP-1 is found to be associated with cancer (88), and NLRP3 can down-regulate TIMP-1 (89). Phytanol and phytanyl amine exerted an immunostimulatory effect through decreasing the expression of TIMP-1 in a mouse model (16). A recent review by Mangan et al. (12) has been published, with a focus on NLRP3 inflammasome biology, activation pathways, and its roles in mammalian pathophysiology, current drug target, and future aspects. Taken all together, it is widely accepted that diterpenes have diverse biological responses in various test systems *via* interaction with the NLRP3 inflammasome.

Benzodiazepines (e.g., DZP) are positive allosteric modulators of the neurotransmitter gamma-aminobutyric acid (GABA_A_) receptor. GABA, the major inhibitory neurotransmitter in the brain, after binding to benzodiazepines, increases the total conduction of chloride ions across the neuronal cell membrane and causes chloride ion influx, hyperpolarizing the neuron's membrane potential. Therefore, the difference between resting potential and threshold potential is increased, and firing is less likely. As a result, arousal of the cortical and limbic systems in the central nervous system is reduced (25). To exert an anxiolytic effect, DZP appears to act on areas of the limbic system, thalamus, and hypothalamus. Plant-derived compounds, including terpenes and terpenoids, possess anxiolytic effects in a wide range of animal models of anxiety (35). In fact, diterpenes are a promising source of neurological agents (90).

The recent human threat SARS-CoV-2 is known to produce cytokine storm in Covid-19 patients. It is evident to release pro-inflammatory cytokines, including IL-6 and IL-1β through the NLRP3 inflammasome pathway (91). Many natural products and their derivatives, including diterpenes, have been found to act against human coronaviruses, such as SARS-CoV and the Middle East respiratory syndrome-related coronavirus (MERS-CoV) (92, 93). Therefore, diterpenes and their derivatives acting through the NLRP3 inflammasome pathway might be a new hope to fight against the current pandemic outbreak.

## Conclusions

At the present time, the utilization of natural products and their derivatives has gained popularity due to the lesser side effects and economy as compared with other treatment strategies. Cumulative research papers have dealt with the health-promoting benefits of diterpenes and their derivatives as they could be used as chemotherapeutic tools against many diseases, including cancer, diabetes, obesity, and neurological diseases and disorders. Findings from this review highlight the role of diterpenes and their interaction with the NLRP3 inflammasome. In addition, this review has shown that NLRP3 inflammasome can be an important molecular platform in the induction of the central pro-inflammatory cytokine IL-1β *via* the activation of CASP1. The NLRP3 inflammasome is able to act through the mitochondria; therefore, it has an important impact on physiological homeostasis and metabolic balance. Furthermore, this review has shed some light on 13 important diterpenes that act through the NLRP3 inflammasome interactive pathway, providing new insights for the development of new therapeutic modalities.

## Author Contributions

MI and JS-R: conceptualization. SB: validation investigation. MI, SB, MM, WK, KG-B, BA, and JS-R: resources. MI, SB, and MM: data curation. MI, JS-R, and WK: review and editing. All the authors writing, read, and approved the final manuscript. All authors contributed equally to the manuscript.

## Conflict of Interest

The authors declare that the research was conducted in the absence of any commercial or financial relationships that could be construed as a potential conflict of interest.

## Abbreviations

NLRP3: NOD-like receptor pyrin domain-containing protein 3
CINCA: chronic infantile neurological cutaneous and articular
FCAS: familial cold auto-inflammatory syndrome
MWS: Muckle–Wells syndrome
NOMID: neonatal onset multisystem inflammatory disease
NSAIDs: non-steroidal anti-inflammatory drugs
Eos: essential oils
NO: nitric oxide
LPS: lipopolysaccharide
MCP-1: monocyte chemoattractant protein-1
VCAM-1: vascular cell adhesion molecule-1
ROS: reactive oxygen species
TXNIP: thioredoxin-interacting protein
PPAR-α: peroxisome proliferator-activated receptor-α
ASK1: apoptosis signal-regulating kinase 1
MAPK: mitogen-activated protein kinase
cNOS: constitutive NO synthase
TNF-α: tumor necrosis factor-alpha
NF-κB: nuclear factor kappa B
CDAA: choline-deficient, l-amino acid defined
HMGP1: high-mobility group protein 1
TLR4: toll-like receptor 4
COX-2: cyclooxygenase-2
TGF-β: tumor growth factor beta
iNOS: inducible nitric oxide synthase
MIP-1α: macrophage inflammatory protein 1-alpha
PKC: protein kinase C
APP: amyloid precursor protein
Nrf2: nuclear factor erythroid 2
MDA: malondialdehyde
SREBP-1: sterol regulatory element-binding protein 1
HMGB1: high-mobility group protein B1
TIMP-1: tissue inhibitor of metalloproteinase-1.
