# Corrigendum: Immunomodulatory Effects of Diterpenes and Their Derivatives Through NLRP3 Inflammasome Pathway: A Review

**DOI:** 10.3389/fimmu.2021.692302

**Published:** 2021-04-22

**Authors:** Muhammad Torequl Islam, Sanaa K. Bardaweel, Mohammad S. Mubarak, Wojciech Koch, Katarzyna Gaweł-Beben, Beata Antosiewicz, Javad Sharifi-Rad

**Affiliations:** ^1^ Laboratory of Theoretical and Computational Biophysics, Ton Duc Thang University, Ho Chi Minh City, Vietnam; ^2^ Faculty of Pharmacy, Ton Duc Thang University, Ho Chi Minh City, Vietnam; ^3^ Department of Pharmaceutical Sciences, School of Pharmacy, The University of Jordan, Amman, Jordan; ^4^ Department of Chemistry, The University of Jordan, Amman, Jordan; ^5^ Chair and Department of Food and Nutrition, Medical University of Lublin, Lublin, Poland; ^6^ Department of Cosmetology, University of Information Technology and Management in Rzeszów, Rzeszów, Poland; ^7^ Zabol Medicinal Plants Research Center, Zabol University of Medical Sciences, Zabol, Iran

**Keywords:** diterpenes, inflammation, mitochondrial dysfunction, NLRP3, mechanism of action

In the published article, there was an error in affiliation 2. Instead of “Faculty of Pharmacy, Ho Chi Minh City, Vietnam,” it should be “Faculty of Pharmacy, Ton Duc Thang University, Ho Chi Minh City, Vietnam”.

The authors apologize for this error and state that this does not change the scientific conclusions of the article in any way. The original article has been updated.

